# Microstructural and Tensile Properties Anisotropy of Selective Laser Melting Manufactured IN 625

**DOI:** 10.3390/ma13214829

**Published:** 2020-10-28

**Authors:** Mihaela Raluca Condruz, Gheorghe Matache, Alexandru Paraschiv, Tiberius Florian Frigioescu, Teodor Badea

**Affiliations:** National Research and Development Institute for Gas Turbines COMOTI, 220D Iuliu Maniu Av., 061126 Bucharest, Romania; gheorghe.matache@comoti.ro (G.M.); alexandru.paraschiv@comoti.ro (A.P.); tiberius.frigioescu@comoti.ro (T.F.F.); teodor.badea@comoti.ro (T.B.)

**Keywords:** microstructure, anisotropy, tensile strength, SLM, IN 625, simulation

## Abstract

The present study was focused on the assessment of microstructural anisotropy of IN 625 manufactured by selective laser melting (SLM) and its influence on the material’s room temperature tensile properties. Microstructural anisotropy was assessed based on computational and experimental investigations. Tensile specimens were manufactured using four building orientations (along *Z, X*, *Y*-axis, and tilted at 45° in the *XZ* plane) and three different scanning strategies (90°, 67°, and 45°). The simulation of microstructure development in specimens built along the *Z*-axis, applying all three scanning strategies, showed that the as-built microstructure is strongly textured and is influenced by the scanning strategy. The 45° scanning strategy induced the highest microstructural texture from all scanning strategies used. The monotonic tensile test results highlighted that the material exhibits significant anisotropic properties, depending on both the specimen orientation and the scanning strategy. Regardless of the scanning strategy used, the lowest mechanical performances of IN 625, in terms of strength values, were recorded for specimens built in the vertical position, as compared with all the other orientations.

## 1. Introduction

In the last decades, extensive research has been made in the field of advanced manufacturing technologies, focusing primarily on additive manufacturing (AM), to understand the process limitations, and develop different methods to overcome its barriers. The development of new complex product designs and the general idea of reducing pollution by using environmentally friendly manufacturing methods has led to an ascending evolution of AM. Many materials are used as feedstock for various AM methods [1], but in terms of metallic parts manufacturing, two categories of metal AM technologies have been developed: powder bed methods (i.e., SLM, electron beam melting—EBM, and direct metal laser sintering—DMLS) and powder/wire fed methods (laser cladding, direct energy deposition, and laser metal deposition). Even if it was invented more than twenty years ago [2,3], SLM has recently attracted increasing attention.

Extensive research has been conducted regarding this technology, pointing out its advantages and disadvantages. The main drawbacks of AM technology are the anisotropy and heterogeneity of materials, in terms of mechanical properties and microstructure [4,5,6,7]. According to the research made by DebRoy et al. [4] and Kok et al. [7], the additive manufactured material’s anisotropic behavior is initiated within the microstructural development, and is influenced by seven factors: grain morphology, crystallographic texture, lack-of-fusion defects, phase transformation, heterogeneous recrystallization, layer banding, and microstructural coarsening. Additive manufactured metals have a complex microstructure in the as-built state, which consists in small equiaxed grains formed near the melt pool boundaries. The grains are characterized by different crystallographic orientations due to rapid cooling, while the large columnar grains nucleate and grow epitaxially near the fusion line, and extend on multiple layers due to partial remelting of previously deposited layers.

These microstructural morphologies are common for many alloys, such as IN 625 and IN 718 Ni-based superalloys [8,9,10,11,12,13,14,15,16,17,18], titanium, or cobalt alloys [19,20,21,22,23,24], and stainless steel [25]. The microstructure’s anisotropic behavior dictates the material’s mechanical properties, thereby the additively manufactured metal’s mechanical properties are defined according to the building orientation [26].

The anisotropic behavior of additive manufactured metals has been investigated in many studies [27,28,29,30] in order to reduce it by various post-processing techniques, like heat treatment optimization or hot isostatic pressing (HIP). The studies conducted on additively manufactured IN 718 Ni-based superalloy showed that its anisotropic behavior depends on the building angle, building orientation, scanning strategy, phase development, texture, and grain morphology [30,31,32,33,34,35].

Contrary to other studies, Jiao et al. [36] studied the elevated-temperature tensile and fatigue performances of K536 Ni-based superalloy manufactured by SLM, and they found that the material does not experience a significant anisotropic behavior.

To reduce production costs and material losses, computational methods have been developed for studying the microstructural evolution during the solidification of conventional metallic materials, and for optimizing their quality characteristics. The last decade’s progress in the field of computational methods was focused on updating the conventional material databases with additively manufactured materials. The main methods used to analyze the microstructural development are cellular automaton (CA), Monte Carlo (MC), Phase-field (PF), Lattice Boltzmann (LB), Molecular dynamics (MD), and other empiric models [37,38,39,40], or combined models, such as CALB [41].

The present study is focused on the assessment of microstructural anisotropy of IN 625 manufactured by SLM, and its influence on the material’s room temperature tensile properties.

## 2. Materials and Methods

For microstructural analysis and tensile tests, prismatic specimens (10 × 10 × 15 mm^3^) and cylindrical coupons 80 mm long and 11 mm in diameter were manufactured, using a DMG MORI, Lasertec 30 SLM machine (DMG MORI, Bielefeld, Germany) and IN 625 metal powder (15–45 μm particle range) produced by LPW Technology Ltd (Runcorn, UK) as feedstock. The powder’s chemical composition is presented in Table 1, and it was characterized by the following powder size distribution: D_10_ = 22 μm, D_50_ = 34 μm, and D_90_ = 42 μm, experimentally determined by the authors [42].

**Figure 1:**
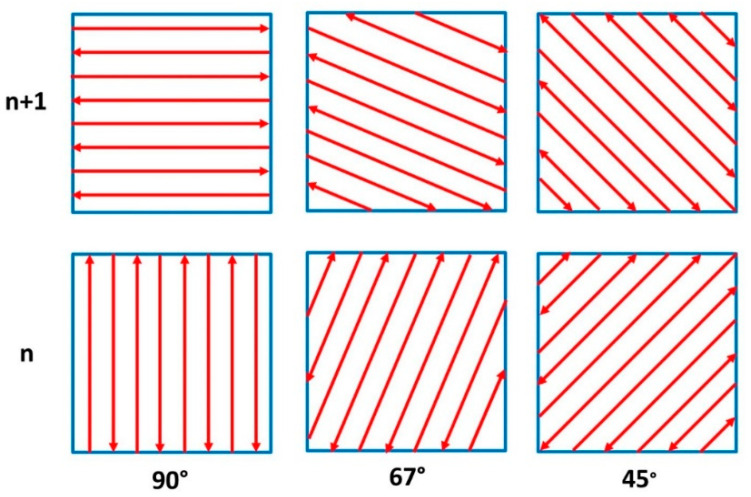
Scanning strategies used in the current study.

All specimens were manufactured on a heated building plate (80 °C) using the same process parameters: 250 W laser power, 750 mm/s laser speed, 40 μm layer thickness, 0.11 mm hatch distance, and 70 µm laser focus. All specimens, for both microstructural analysis and tensile testing, were manufactured using three different scanning strategies, respectively a 45°, 67°, and 90° scanning strategy, rotated by 90° between two successive layers, as schematically shown in Figure 1. The difference between the three scanning strategies used (45°, 67°, and 90°, all with a 90° change of the scanning direction between successive layers) is represented by the angle of the laser path.

**Figure 2:**
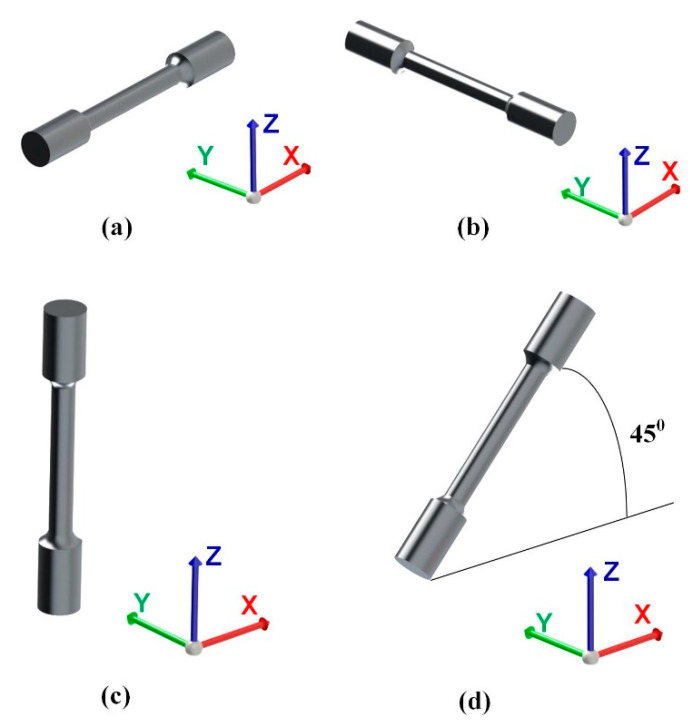
Specimens’ building orientation: (**a**) *X*-axis; (**b**) *Y*-axis; (**c**) *Z*-axis; (**d**) tilted at 45° in the *XZ* plane.

**Figure 3:**
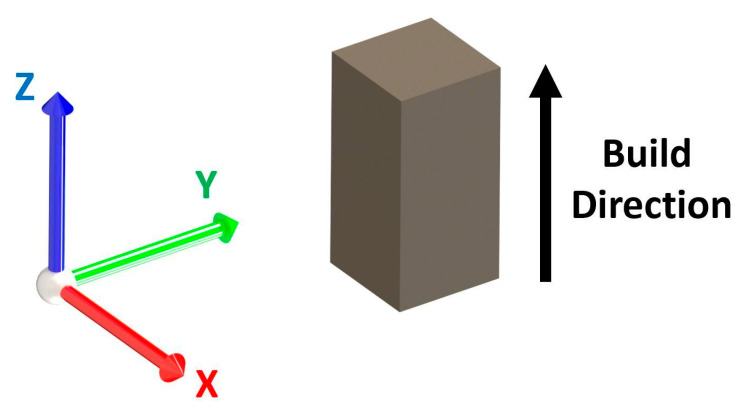
Prismatic specimen intended for microstructural analysis.

The coupons intended for machining tensile test pieces were manufactured on four different building orientations: along *X, Y, Z*-axis, and tilted at 45° in the *XZ* plane, as depicted in Figure 2, while the prismatic specimens used for microstructural analysis were built in a vertical position (Figure 3). In all cases, and henceforth in this study, the *X*-axis is parallel to the front of the machine, while the *Z*-axis designates the vertical direction.

Two sets, of seven cylindrical coupons each, were manufactured for each building orientation and each scanning strategy. Two prismatic specimens were manufactured using each of the three scanning strategies. One of the specimens was used for microstructural analysis in the as-built condition, while the second was heat treated together with the coupons.

**Figure 4:**
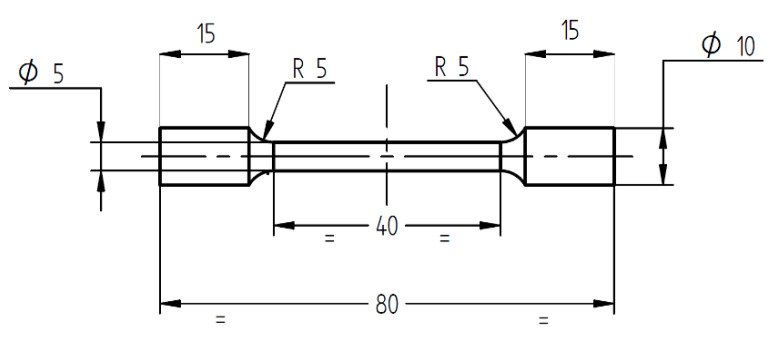
Geometry and dimensions of the tensile test pieces (units in millimetres).

All specimens and coupons were subjected to heat treatment in air using an electrical Nabertherm LH 30/14 chamber furnace (Nabertherm GmbH, Lilienthal, Germany). The heat treatment consisted of stress relieve heat treatment (heating from room temperature until 870 °C, holding for 1 h, and cooling in air to room temperature), and annealing heat treatment (heating from room temperature until 1000 °C, holding for 1 h followed by fast cooling (oil quenching)). The heat treatment regimen used was adapted for AMed IN 625; starting from the typical heat treatment of conventionally manufactured IN 625, the same stress relieving temperature was used, but the temperature for the annealing heat treatment was increased by 20 °C for the AMed IN 625, and the cooling was realized in oil not in water [43]. Standard round tensile test pieces were machined from annealed coupons, according to the geometry and dimensions presented in Figure 4.

Monotonic tensile tests were performed at room temperature according to ISO 6892-1:2009 using an electromechanical universal testing machine, Instron 3369 (Instron, Norwood, MA, USA), equipped with a 50 kN load cell. During the tensile test, the strain rate over the parallel length was set to ${\dot{e}}_{Lc}$ = 0.00025 s^−1^ until the detection of 0.2% yield strength, then the extensometer was removed and the strain rate over the parallel length was set to ${\dot{e}}_{Lc}$ = 0.0067 s^−1^. The tensile properties’ anisotropy was expressed as a function of the values recorded on the *Z*-axis specimens, using Equation (1) [44].

(1) $$\frac{\sigma_{i1}-\sigma_{i2}}{\sigma_{i1}}\;\cdot 100$$

where *σ_i_*_1_ is the average ultimate tensile strength (UTS)/0.2% yield strength (YS)/elongation/reduction of area value obtained on test pieces manufactured along *X*-axis or tilted at 45° in the *XZ* plane. *σ_i_*_2_ is average UTS/0.2% YS/elongation/reduction of area value obtained on test pieces manufactured in vertical position, along *Z*-axis, that showed the lowest strength values.

Microstructural simulations were performed using the ANSYS Additive Suite (ANSYS, Inc., Canonsburg, PA, USA), Additive Science module, R1/2020 edition. Due to software limitations, the simulations were realized only on specimens manufactured on the *Z*-axis by applying all three scanning strategies, and the same manufacturing process parameters as in the case of the experimental procedure, except the laser focus, which for the simulation was 80 µm (the lowest value that can be applied).

For microstructural analysis by finite element cellular automaton method, the IN 718 was selected from the material database as currently, it is the only Ni-based superalloy available in the ANSYS Additive Suite database (ANSYS, Inc., Canonsburg, PA, USA) validated for microstructural prediction. The software was developed for IN718 alloy which belongs to the same Ni-Cr superalloys class with the investigated IN 625 alloy. As the software allows the customization of input data, the simulation was done using the actual experimental process parameters used for IN 625 in the current study.

The simulation analysis showed the microstructure evolution as a 1 mm^2^ surface of the *XY*, *XZ*, and *YZ* planes. The experimental microstructural analysis was performed by scanning electron microscopy (SEM) using an FEI F50 Inspect (FEI Company, Brno, Czech Republic) and optical microscopy using the microscope, Axio Vert.A1 MAT (Carl Zeiss Microscopy GmbH, Jena Germany) with camera (Nikon Digital Microscope Camera DS-Fi3, (Nikon Instruments Inc., Melville, NY, USA), and NIS-Elements software (version 5.02.03, Nikon Instruments Inc., Melville, NY, USA).

Microstructural analysis was performed on metallographically prepared prismatic specimens by grinding, polishing, and etching with Aqua Regia for 20 s. For the grain size measurement the intercept method was used. Optical micrographs captured at 100× magnification were processed using the Scandium software (version 5.2, Olympus Soft Imaging Solutions GmbH, Münster, Germany) by highlighting the grain boundary (red color separation, edge enhance filter, unsharp mask filter) and applying a grid consisting in 5 vertical and 5 horizontal equally spaced lines. The average grain size was determined based on measurements realized on 4 different light optical microscopy images.

Density measurements were made using the Archimedes method, according to ISO 3369 [45], and using an analytical balance, Pioneer PX224 (Ohaus Europe GmbH, Nänikon, Switzerland), with a density kit for solids. The relative density was expressed as the percentage of the ratio between the average of 5 measurements made on a prismatic specimen for each scanning strategy used, and the material’s theoretical density calculated based on its chemical composition. The auxiliary liquid used for measurements was ethanol (99.3% purity), and all specimens were degreased before testing using the same alcohol. The measurements were made at 20 °C room temperature, and 19.9 °C ethanol temperature.

## 3. Results

### 3.1. Microstructural Analysis

**Figure 5:**
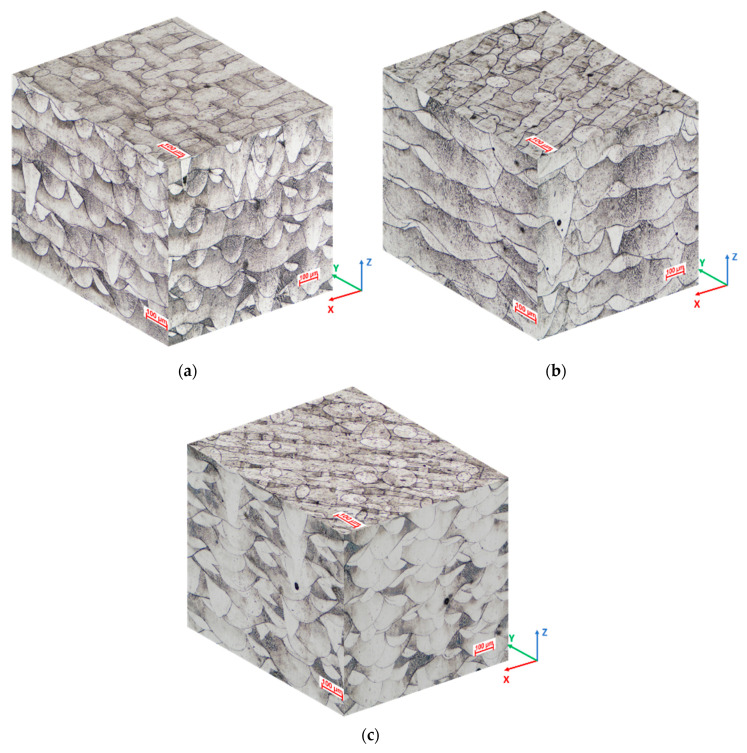
Representative 3D reconstruction of the as-built IN 625 microstructure: (**a**) 90° scanning strategy; (**b**) 67° scanning strategy: 100× magnification; (**c**) 45° scanning strategy.

In the as-built state, the SLM manufactured IN 625 microstructure reveals particular characteristics on different orientations, determined by the layer by layer building process. The layer boundaries and the melt pool trace are typical features, as can be observed in the 3D microstructure reconstruction of a specimen manufactured using a 90° scanning strategy, presented in Figure 5.

**Figure 6:**
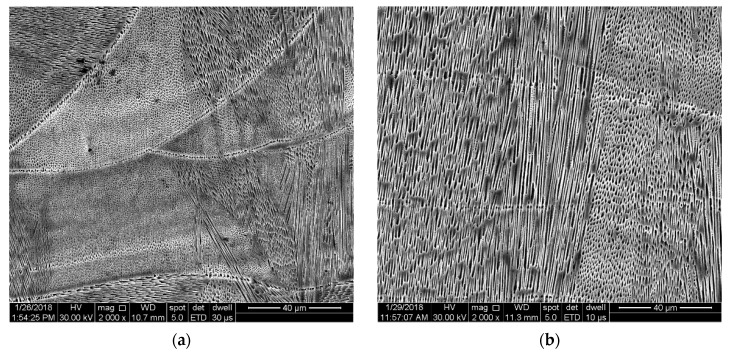
SEM images revealing microstructural features of as-built IN 625 (90° scanning strategy: *XZ* plane) (**a**) layer boundaries with different columnar dendritic and cellular morphology arrays and (**b**) columnar grains that exceed the layer thickness.

It was noticed that in the as-built state, even after etching, the grain boundaries were not as visible as in the case of other conventionally manufactured materials. SEM micrographs presented in Figure 6 highlight the heat-affected zone (HAZ) and the microstructure morphology in the *XZ* plane. A particular feature of the microstructure is the columnar dendritic array that exceed the layer thickness.

**Figure 7:**
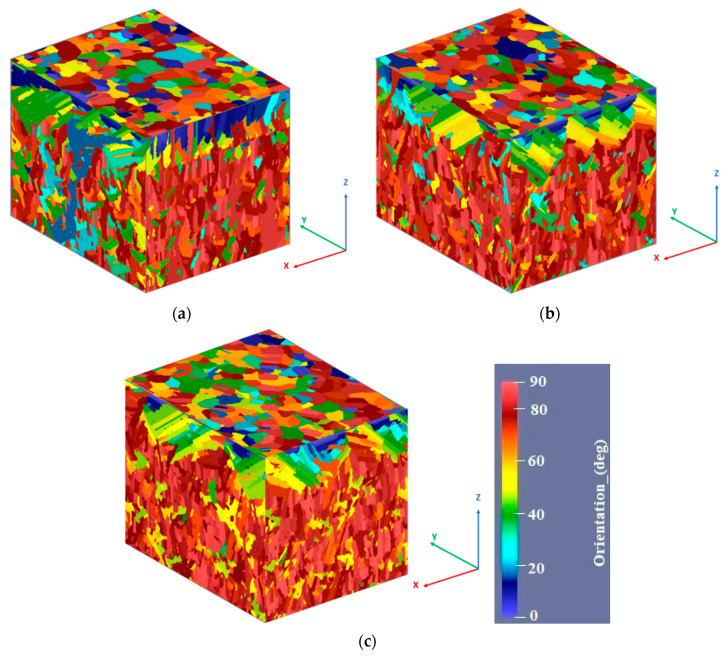
3D microstructure reconstructions based on microstructural simulation results of specimens manufactured along the *Z*-axis using scanning strategies at (**a**) 90°, (**b**) 67°, and (**c**) 45°.

The results of the finite element microstructural simulation are shown in the 3D reconstructions from Figure 7. The as-built microstructure is characterized by a substantial texture that is influenced by the scanning strategy. The texture degree increases as the scanning strategy angles are reduced from 90° to 45°.

**Figure 8:**
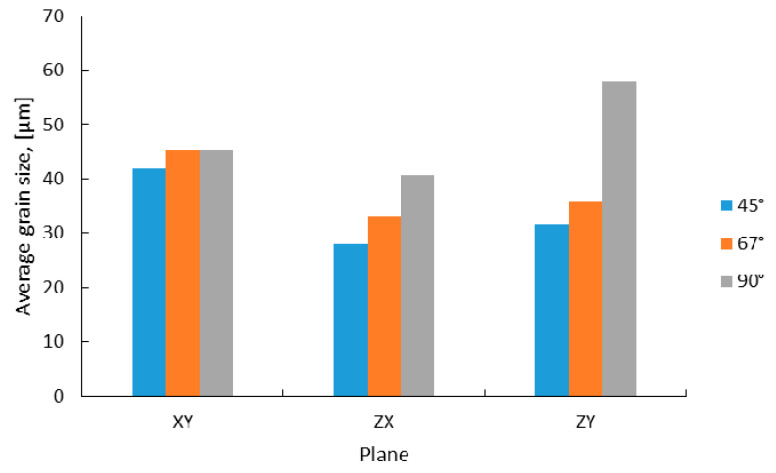
Average grain size predicted by simulation.

Using a 90° scanning strategy, long columnar grains developed along the *Z*-axis in *XZ* and *YZ* planes across several layers as a result of material partial remelting during multiple laser passes, while in the *XY* plane equiaxed grain growth was predicted (Figure 7a). Long columnar grains were also predicted for specimens manufactured by applying a 67° scanning strategy, but they are not as long as the grains developed in the material manufactured using a 90° scanning strategy (Figure 7b). The 45° scanning strategy generates the highest microstructural texture of all the scanning strategies used (Figure 7c). The average grain size predicted by the computational method as a function of the scanning strategy in different planes is presented in Figure 8.

Figure 8 shows that the 90° scanning strategy resulted in coarser grains as compared with the other scanning strategies, while the most refined microstructure was predicted by applying a 45° scanning strategy, regardless of the plane where the microstructure was analyzed. This behavior is caused by the differences in the cooling rate (CR) and thermal gradient (G).

The software predicted a G value of 4.4 × 10^6^ K/m and a CR value of 4.7 × 10^5^ K/s for the specimens manufactured using a 90° scanning strategy. By changing the scanning strategy, an increase in both G and CR values was registered. As the angle of the scanning strategy was reduced, a progressive increase was obtained, reaching the highest G value of 5.6 × 10^6^ K/m and a CR value of 5.2 × 10^5^ K/s for the specimens manufactured using a 45° scanning strategy.

**Figure 9:**
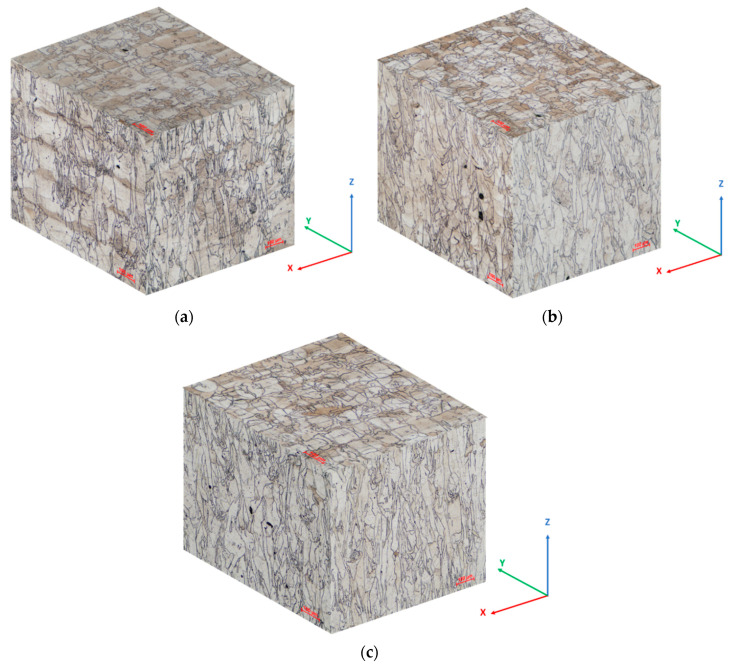
3D microstructure reconstructions for the annealed specimens manufactured along *Z*-axis using the scanning strategies at (**a**) 90°; (**b**) 67°, and (**c**) 45°: experimental results.

The finite element simulation provides results regarding the microstructure of additive manufactured materials in the as-built state, but similarities were observed between the images generated by the software and the microstructural investigation performed on IN 625 in an annealed state (Figure 9).

**Figure 10:**
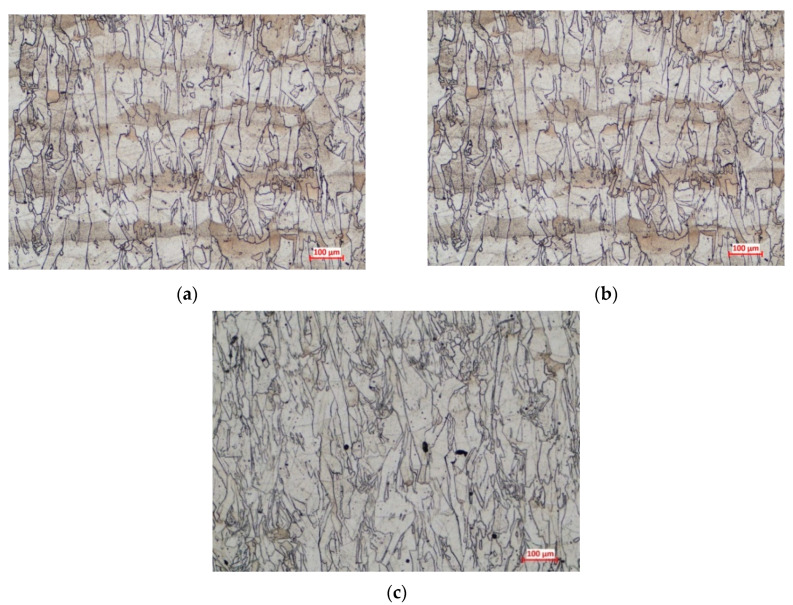
Optical micrographs taken in the *XZ* plane of the annealed additively manufactured (AM) IN 625 produced with scanning strategy at (**a**) 90°, (**b**) 67°, and (**c**) 45°.

**Figure 11:**
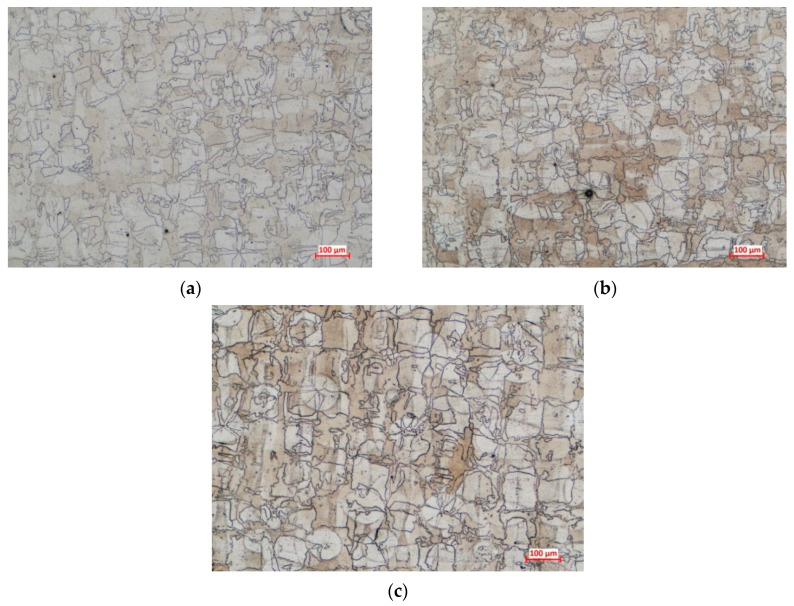
Optical micrographs taken in the *XY* plane of the annealed AM IN 625 produced with scanning strategy at (**a**) 90°, (**b**) 67°, and (**c**) 45°.

The applied heat treatment stimulated the grains’ recrystallization, but the as-built microstructural texture was still retained even after recrystallization. Columnar grains were identified in the *XZ* and *YZ* planes, while equiaxed grains were observed in the *XY* plane on all specimens, as was also predicted by the finite element analysis. Moreover, it was observed that the annealing heat treatment caused the formation of annealing twins, as can be seen in the optical micrographs presented in Figure 10 and Figure 11. Annealing twins were observed at a higher extent in the case of specimens manufactured using 67° and 45° scanning strategies.

**Figure 12:**
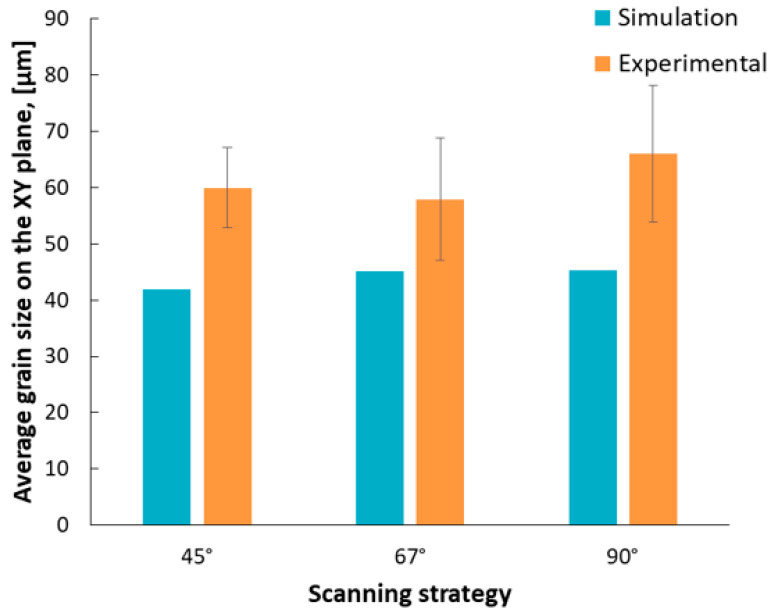
Predicted and measured grain size in *XY* plane as a function of scanning strategy.

The average grain size was measured in the *XY* plane, for specimens manufactured using all scanning strategies. The results obtained in the case of dimensional analysis of equiaxed grains were compared with the simulation results obtained in the same plane. The average measured, and predicted, grain size are presented in Figure 12, showing that the 90° scanning strategy results in coarser grains compared with the other two scanning strategies.

### 3.2. Density Measurements

The 67° scanning strategy generated the highest relative density (99.39%) compared with the values recorded for the specimens manufactured using the 90° scanning strategy (99.37%) and 45° scanning strategy (99.34%).

### 3.3. Tensile Testing

**Figure 13:**
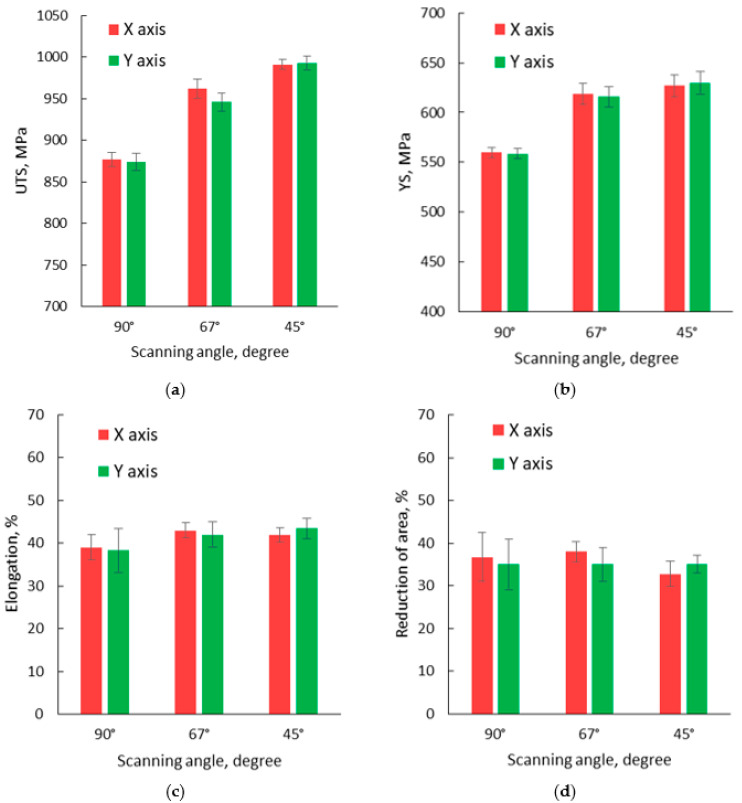
Tensile test results obtained for specimens manufactured on the *XY* plane: (**a**) ultimate tensile strength (UTS); (**b**) yield strength (YS); (**c**) elongation; and (**d**) reduction of area.

Monotonic tensile tests were performed at room temperature on all specimen sets built with different orientations and scanning strategies. The average tensile test results of all 14 samples produced for each orientation and scanning strategy are presented in Appendix A. The specimens manufactured along the *X*-axis and *Y*-axis using all three scanning strategies were evaluated with respect to tensile properties in a horizontal position (UTS, 0.2% YS, elongation and reduction of area). The tensile test results and standard deviations for all specimens manufactured on the *XY* plane are presented in Figure 13.

**Figure 14:**
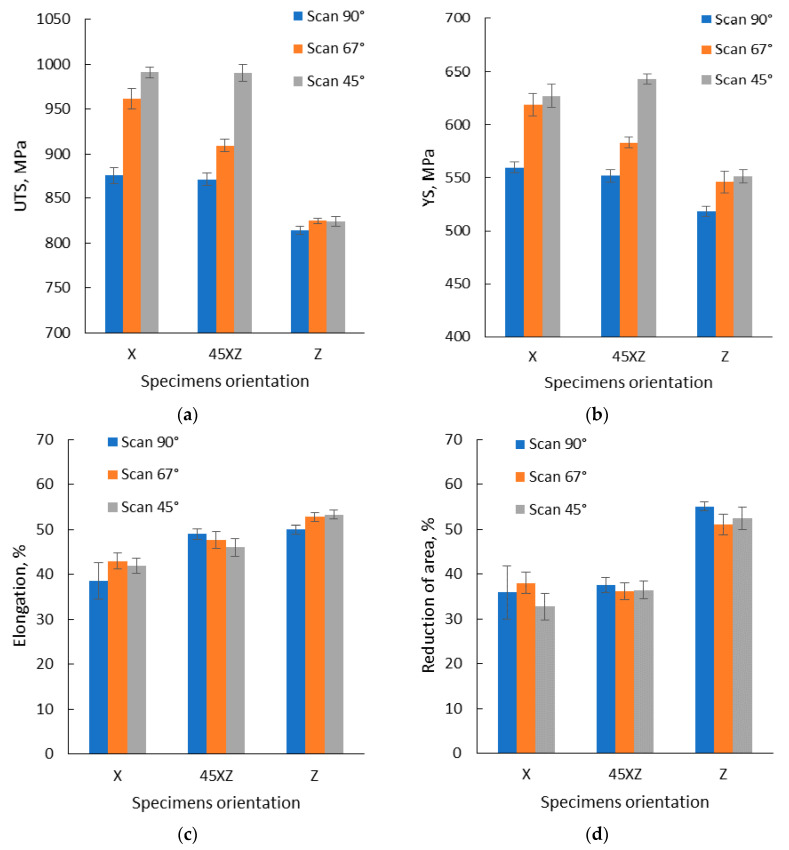
Tensile test results obtained for specimens manufactured on three different orientations and scanning strategies: (**a**) UTS; (**b**) YS; (**c**) elongation and (**d**) reduction of area.

**Figure 15:**
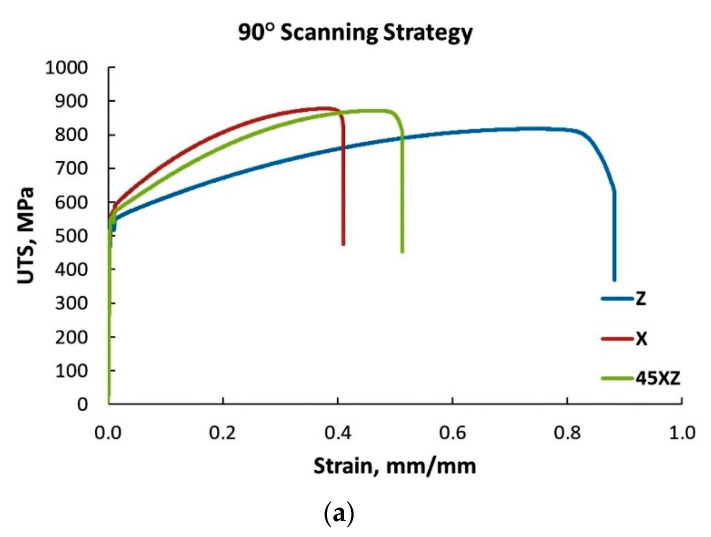

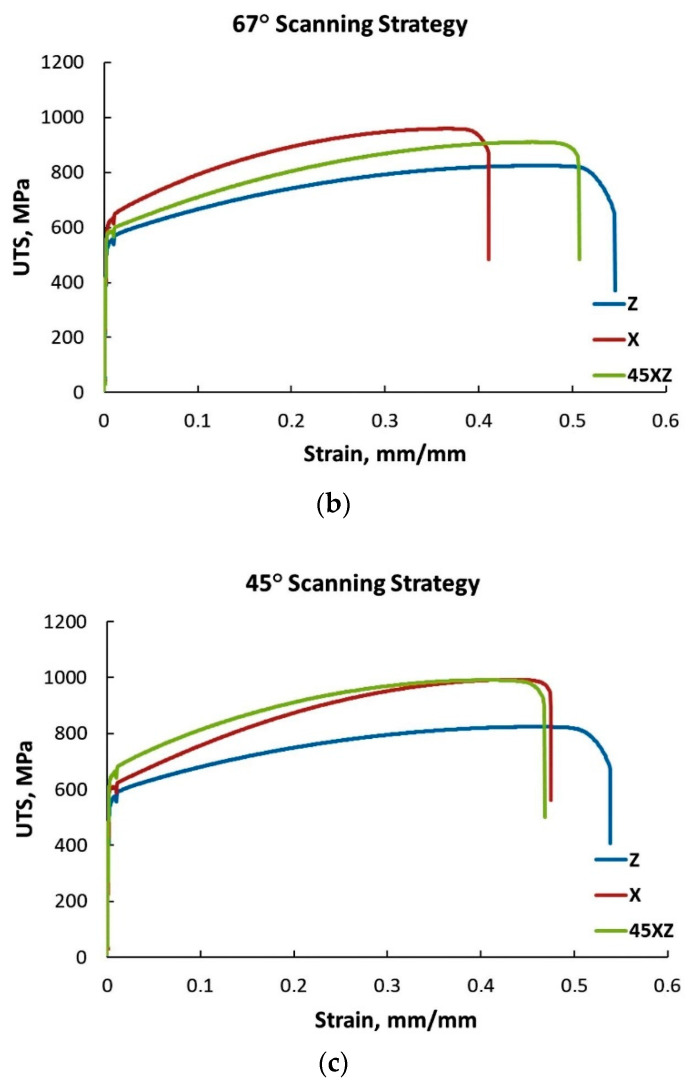
Representative stress–strain curves as a function of the scanning strategy at: (**a**) 90°; (**b**) 67° and (**c**) 45°.

Based on the tensile test results, presented in Figure 13, it was concluded that similar or closed values were recorded for specimens manufactured along the *X* and *Y*-axis using the same scanning strategy. The highest tensile strength was recorded for specimens manufactured using a 45° scanning strategy, while the lowest tensile strength was recorded for specimens manufactured using a 90° scanning strategy. The same evolution was observed for the yield strength, but no significant differences were recorded for the specimen’s elongation and reduction of area (Table A1), and they don’t follow a specific trend line. As no significant differences were recorded between specimens manufactured in the *XY* plane, along *X,* or *Y*-axis, to determine the influence of building orientation and scanning strategy on the tensile properties only the *X*-axis specimens’ test results were used further in the analysis. The average tensile test results on test pieces built with different orientations and scanning strategies are presented in Figure 14, and a representative set of stress–strain curves is presented in Figure 15.

The monotonic tensile test results highlighted that IN 625 manufactured by SLM exhibits significant anisotropic tensile properties, depending on the specimen orientation. Regardless of the scanning strategy, the lowest strength (both UTS and YS) was recorded for the specimens built along the *Z*-axis. On the contrary, for all scanning strategies, the test pieces manufactured along the *Z*-axis exhibited higher elongation at fracture, and much higher reduction of area as compared with the other two building orientations.

Based on the tensile test results, it can be noticed that even after the heat treatment, the microstructural anisotropy generated by the manufacturing process causes the anisotropic tensile behavior of the alloy. Table 2 presents the anisotropy of tensile properties calculated using Equation (1) for SLM manufactured IN 625 as a function of tensile test results of the building orientation that exhibited the lowest strength, i.e., test pieces built in the vertical position.

## 4. Discussion

The present study was focused on the assessment of microstructural anisotropy of SLM manufactured IN 625 and its influence on the material’s room temperature tensile properties. Microstructural anisotropy was assessed based on computational and experimental investigations.

The light optical microscopy investigation showed that the SLM manufactured IN 625’s microstructure had similarities with the microstructure of welds, showing a fish scale morphology in the *XZ* and *YZ* planes, where layer boundaries are visible, and in the *XY* plane, where the melt pool trace can be observed. Cellular and columnar dendritic arrays were noticed in multiple specimen sections. Many other authors have observed melt-pool bands, micro-cellular, and columnar arrays in additively manufactured IN 625 [8,10,13,14,26,46,47].

The final solidification microstructure and the crystallographic texture of AM materials are controlled by the process parameters that affect the G and CR values, factors that determine the morphological features. Different values of G and interface growth (R) result in different features of the resulted microstructure that can be planar, cellular, columnar dendritic, and equiaxed dendritic [48].

**Figure 16:**
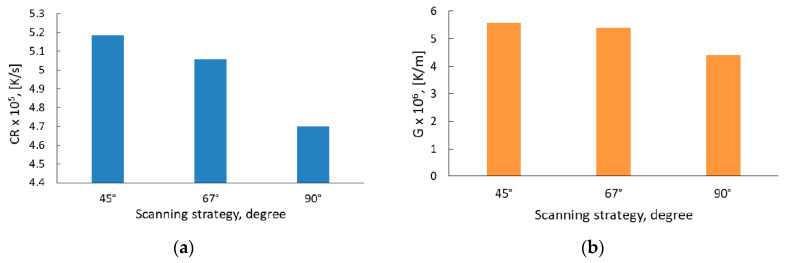
Scanning strategy influence on the (**a**) cooling rate and (**b**) thermal gradient: results predicted by simulation.

In the current study, the ANSYS model predicted that the G and CR values are influenced by the scanning strategy. The 90° scanning strategy results in a G value of 4.4 × 10^6^ K/m, and an CR value of 4.7 × 10^5^ K/s. By reducing the angle of the scanning strategy from 90° to 45° a progressive increase in the G and CR values was recorded, reaching a G value of 5.6 × 10^6^ K/m and CR value of 5.2 × 10^5^ K/s for specimens manufactured using a 45° scanning strategy. The values obtained have the same order of magnitude as the values recorded by Letenneur et al. [49] and Gan et al. [50], when similar process parameters were used for IN 625. The scanning strategy influence on the CR and G is presented in Figure 16.

In the as-built state, ANSYS predicted a textured microstructure parallel to the building direction that also depends on the scanning strategy. Textured microstructure was also identified by Risse et al. [51] for IN 738LC, while Gonzalez et al. [26] reported textured columnar microstructure perpendicular to the build direction of IN 625 manufactured by EPBF; 16 µm wide and 356 µm long columnar grains being measured. Fine equiaxed and columnar grain development was also predicted by Lian et al. [52] for IN 718 using a 3D cellular automation finite volume method.

It is assumed that heat treatment post-processing can be used as a method to homogenize the microstructure of additive manufactured materials [53,54]. For example, Marchese et al. [8] applied different heat treatment regimens to additively manufactured IN 625, and observed that a solution treatment at 1150 °C for 2 h led to material recrystallization, and different grain sizes were obtained. The resulting microstructure was comprised large grains around 90 µm, and also 10 µm fine grains.

In the present study, average grain size between 28–58 µm was predicted as a function of the plane where the grains developed and the scanning strategy. The 90° scanning strategy produced the highest average grain size, regardless of the plane where they developed, while the 45° scanning strategy produced the lowest average grain size. Using similar process parameters, the computational model developed by Letenneur et al. [49] for IN 625 predicted a different grain size (100 µm on the plane *XZ*), which was in good agreement with the experimental results of Poulin et al. [55].

Comparing the results predicted by ANSYS (ANSYS, Inc., Canonsburg, PA, USA) for equiaxed grains (*XY* plane), in the as-built state, with the results experimentally determined for the same grains in heat treatment state, a similar dimensional evolution was observed. The 90° scanning strategy led to the formation of larger grains (45 µm by computational method, respective to 66 µm experimentally measured), as compared with smaller grains resulted in specimens manufactured with the 45° scanning strategy (41 µm, and 60 µm, respectively).

It was predicted that in the as-built state in the *XZ* and *YZ* planes, long columnar grains would grow parallel to the building direction. These columnar grains that grow over several layers were also observed after the annealing heat treatment, the microstructural texture being pronounced even after post processing. It has been experimentally measured that these columnar grains can reach lengths up to 750 µm for the material manufactured with a 90° scanning strategy.

A distinctive feature of annealed conventional manufactured face-centered cubic (FCC) materials is the formation of annealing twins [56,57]. Such a microstructural feature was highlighted in this study by light optical microscopy for SLM manufactured IN 625. Annealing twins were also observed by other authors for IN 625 produced using other additive manufacturing methods, i.e., binder jetting, and electron beam melting [28,58].

So far, many studies have been conducted regarding the formation and evolution of annealing twins in FCC materials [59,60,61], and four theories have been proposed to sustain the formation of twins: the growth accident [58], the dissociation of the grain boundary [59], grain encountering during recrystallization [60], and coalescence of stacking fault packets nucleating at grain boundaries [61]. Based on twin topologies, Bozzolo et al. [62] divided the annealing twins into grain growth twins and recrystallization twins. Jin et al. [63] conducted studies on the twin density evolution in case of conventional manufactured IN 718, and they maintained that the recrystallization twins are controlled by the propagation of the pre-existing twins of growing grains. As AM IN 625 is characterized by a great microstructural texture in the as-built state, the presence of growing twins is possible and it could influence the density of recrystallization twins. Moreover, the recrystallization twins can form during the heat treatment, as the material releases the residual stress accumulated during manufacturing [64]. Recrystallization twins were also observed by Cao et al. [65] in AM IN, as an effective way to refine the recrystallized grain size.

It is known that the AM process parameters have a great influence on the material’s characteristics, including the relative density. For example, Gao et al. [66] obtained a 99.7% relative density for electron beam selective melting manufactured IN 625, while Terris et al. [67] obtained relative densities in the range of 95.5–99.6% for SLM manufactured IN 625, using different volumetric energy densities (VED). The main parameters that affect the relative density of the AMed materials are the laser power, scanning speed, layer thickness, and hatch spacing (parameters used to calculate the VED value). As in the present study the same process parameters were used, excepting the scanning strategy, it was concluded that the scanning strategy did not have a great influence on the material’s density, as relative densities between 99.34–99.39% were experimentally obtained.

As the microstructural anisotropy of SLM manufactured IN 625 was identified in both as-built and heat-treated material, its influence on the tensile properties was evaluated. Based on the results obtained it was concluded that both scanning strategy and building orientation have a strong influence on the tensile properties. The highest UTS was obtained on specimens manufactured in the *XY* plane, and tilted at 45° in the *XZ* plane, applying a 45° scanning strategy. The lowest UTS was obtained in the case of specimens manufactured along the *Z*-axis, regardless of the scanning strategy used. The same tendency was determined for the material’s YS. In terms of elongation and reduction of area, the specimens manufactured along the *Z*-axis presented higher elongation at fracture and reduction of area, as compared with the results for the other building orientations. The strength increase as the orientation angle decreases was also observed by Anam [68].

Despite the fact that the material is characterized by a considerable anisotropic behavior, the SLM manufactured IN 625’s tensile properties exceed the specification minimum requirements for both conventional (according to ASTM B 443 [69]) and additive manufactured material (according to ASTM F3056-14 [70]) in all building orientations.

Compared with the results obtained in the present study, a higher tensile strength was reported by Witkin et al. [71] for SLM manufactured IN 625 along the *Z*-axis, but only after additional post-processing by hot isostatic pressing. Instead, Foster et al. [72] obtained average tensile strength around 830 MPa for the *Z*-axis manufactured IN 625 by direct metal deposition (DED), which is consistent with the results obtained in the current study. Moreover, Foster et al. [72] obtained an average tensile strength of 890 MPa for the *X*-axis manufactured specimens, which is comparable to the result obtained in the present study for the specimens manufactured by applying a 90° scanning strategy, while higher values were recorded when other scanning strategies were used.

The anisotropy of the tensile strength of the specimens built in different orientations can be explained here in connection with the loading direction relative to layer-by-layer manufacturing process and the induced grain texture. In SLM process, the powder bed and the laser melting track are always parallel to the building plate in the *XY* plane, so that the positioning of the parts in any other orientations in the building volume result in various grain growth orientations relative to the coordinate system.

**Figure 17:**
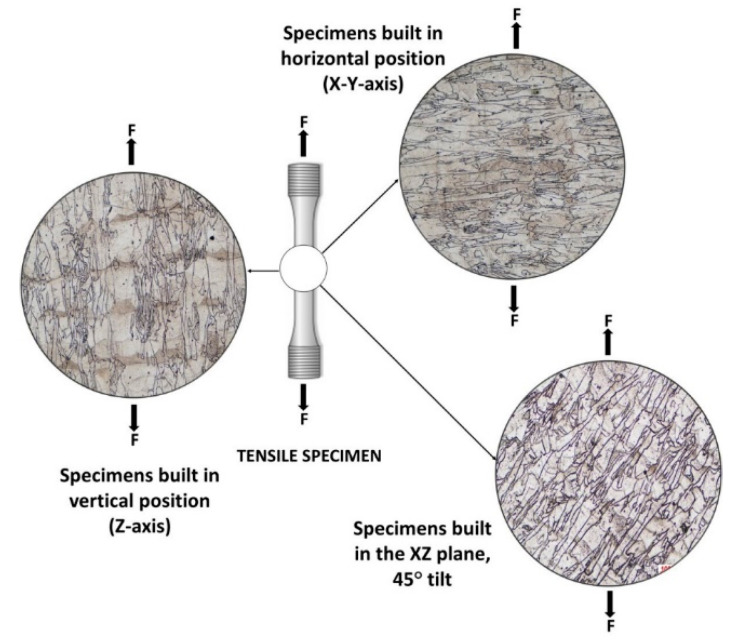
Loading direction (F) of the tensile test pieces relative to the grain growth direction. Micrographs of annealed specimens.

Figure 17 presents schematically the tensile loading direction (F) of the test pieces relative to the grain growth direction for specimens manufactured along the *Z*-axis, in the *XY* plane, and tilted at 45° in the *XZ* plane.

When loading normal to the building direction (specimens built in the *XY* plane) higher yield strength and ultimate tensile strength are recorded, but lower elongation and reduction of area are observed than that when parallel to the building direction (along the *Z*–axis). These anisotropic mechanical properties of AM metallic parts have been reported by several researchers.

The loading direction for specimens built in a vertical position is transverse to a higher number of built layers. Due to the presence of the defects inherently induced by the process between successive layers the load-bearing cross-section is reduced, which explains the lower strength.

For the specimens manufactured in the *XY* plane the load is applied perpendicular to the columnar grains’ growth direction, along the built layers, while in the case of specimens manufactured in a tilt position in the *XZ* plane, the loading is applied at 45° from the growth direction of columnar grains.

If the strength is associated with the loading direction relative to the direction of layer deposition, the higher ductility of the specimens built in vertical position is associated with the long columnar grains grown over multiple layers. Ni et al. [33] showed that the anisotropy of ductility in AM IN 718 is controlled by the different cracking mechanisms induced by the tensile load direction relative to the grain boundaries. They showed that tensile loading perpendicular to the columnar grain-boundary, which corresponds to specimens built in the *XY* plane, complies with Mode I crack opening tension, and leads to lower ductility as compared with loads parallel to the columnar grain boundaries (specimens built along the *Z*-axis). Higher ductility in the latter case is explained by the fact that there are fewer short axes of the grains that comply to Mode I opening tension, and thus the opening failure is more difficult.

For intermediate building orientation (specimens manufactured at 45° in the *XZ* plane), the specimen’s ductility is higher than the ductility of specimens manufactured in the horizontal position, but lower than the ductility of specimens manufactured in the vertical position, as the tensile loading is applied at 45° from the growth direction of columnar grains.

The experimental results of this study are in disagreement with the findings of Yadroitsev et al. [73] who showed that scanning angle variation (0°, 90°, 45°) does not have a significant influence on the UTS and YS of SLM manufactured IN 625 built in a vertical and horizontal position. The experimental results showed that only the tensile strength of the specimens manufactured along the *Z*-axis is not influenced by the scanning strategy. This result can be explained by the length of the scanning line which was not influenced by the scanning strategy, as the specimen’s cross-section was circular.

For the other orientations, the specimens built in the *XY* plane and tilted at 45° on the *XZ* plane, the scanning strategy has a significant influence on the material’s tensile performance. As the scanning angle increases, the UTS and YS decrease. The specimen’s elongation and reduction of area behavior does not depend on the scanning strategy as much as the tensile strength and yield strength, but a slight increase in the elongation was registered for the specimens’ tilted at 45° in the *XZ* plane as the scanning angle increases. The most pronounced anisotropic behavior was registered for the specimens manufactured using the 45° scanning strategy, built in the *XY* plane and in *XZ* plane, associated with the highest microstructural texture level.

## 5. Conclusions

The assessment of SLM manufactured IN 625 anisotropy was studied in terms of microstructure and room temperature tensile properties. To evaluate the microstructural anisotropy, computational and experimental examinations were realized on the *Z*-axis manufactured specimens, applying three scanning strategies (90°, 67°, and 45°). The anisotropic mechanical behavior of the material was evaluated based on specimens manufactured on four-building orientations (along the *Z*, *X*, *Y*-axis, and tilted at 45° in the *ZX* plane) and all three scanning strategies.

By the finite element cellular automaton method the features of the as-built microstructure were determined. It is characterized by a strong texture that depends on the scanning strategy. Using a 90° scanning strategy, in the *XZ* and *YZ* planes long columnar grains develop, distributed over multiple layers, as a result of material partial remelting during multiple laser passes, while in the *XY* plane equiaxed grains were observed. Long columnar grains also formed on specimens manufactured by applying a 67° scanning strategy, but to a lower magnitude. The 45° scanning strategy induces the highest level of microstructural texture from all scanning strategies. The microstructural anisotropy was experimentally determined, and the results obtained in the case of heat-treated specimens were in a good agreement with the computational results.

The monotonic tensile tests performed on annealed test pieces highlighted that SLM manufactured IN 625 exhibits significant anisotropic properties, depending on the specimen orientation and the scanning strategy. The lowest mechanical performances of IN 625 were determined in specimens built in the vertical position, along the *Z*-axis. Even if the material is characterized by considerable anisotropic behavior, it was concluded that the SLM manufactured IN 625 exceeds the specification minimum requirements for both conventional and additive manufactured material.

The general conclusion of the study is that the SLM manufactured IN 625’s microstructural anisotropic behavior led to the anisotropy of mechanical properties, even after heat treatment post-processing.

## Figures and Tables

**Table 1:** Chemical composition of IN 625 metal powder provided by LPW Technology Ltd.

Chemical Elements	Al	C	Co	Cr	Fe	Mn	Mo	Nb	Si	Ti	Ni
Specification [%wt.]	<0.4	<0.1	<1.0	20–23	3–5	<0.5	8–10	3.15–4.15	<0.5	<0.4	Bal.
Test certificate [%wt.]	0.06	0.02	0.1	20.7	4.1	0.01	8.9	3.77	0.01	0.07	62.26

**Table 2:** Anisotropy of tensile properties for different building orientations and scanning strategies.

Scanning Strategy	90°		67°		45°	
Building Orientation	*X*-Axis	Tilt at 45°*XZ*	*X*-Axis	Tilt at 45°*XZ*	*X*-Axis	Tilt at 45°*XZ*
UTS	7.2	6.5	14.3	9.4	16.8	16.8
YS	7.5	6.2	11.8	6.3	12.0	14.2
Elongation	−28.2	−2.0	−22.8	−10.5	−27.2	−15.8
Reduction of area	−49.6	−46.4	−34.2	−41.1	−59.9	−44.0

## Appendix A

**Table A1:** Monotonic tensile test results.

Build Orientation	Scanning Strategy	No. of Specimens	Ultimate Tensile Strength, MPa		0.2% Yield Strength, MPa		Elongation at Rupture, %		Reduction of Area, %	
			Average	STDEV	Average	STDEV	Average	STDEV	Average	STDEV
*X*–Axis	45°	14	991	6	627	11	42	2	33	3
	67°	14	962	11	619	11	43	2	38	2
	90°	14	877	8	560	5	39	3	37	6
*Y*–Axis	45°	14	993	9	630	9	43	2	35	2
	67°	14	946	11	616	11	42	3	35	4
	90°	14	874	10	559	5	38	5	35	6
*Z*–Axis	45°	14	824	5	551	6	53	1	52	2
	67°	14	825	3	546	10	53	1	51	2
	90°	14	814	4	518	5	50	1	55	1
Tilt at 45° in the *XZ* Plane	45°	14	990	9	643	5	46	2	36	2
	67°	14	910	7	583	5	48	2	36	2
	90°	14	870	7	551	6	48	1	38	2
